# Dr. Leslie’s Address on Metallurgy

**Published:** 1849-10

**Authors:** 


### DR. LESLIE'S ADDRESS ON METALLURGY.

DELIVERED BEFORE THE MISSISSIPPI VALLEY ASSOCIATION OF DENTAL SURGEONS, AT ITS FIFTH ANNUAL MEETING, HELD IN LOUISVILLE, 11TH AND 12TH OF SEPTEMBER, 1849.

If we examine the discoveries and advances made in chemical science and those branches which are collateral to it, one thing is made apparent to the most casual observer, and this is the incalculable value of chemistry to the metallurgist. And not only he, but the worker of any one of the metals, from the lowest to the highest. It is chemistry shows best how to unite iron. It is the same science gives us the true theory and correct practice for the union of gold. It takes the rough ore and gives the cheapest mode for its reduction. It extends the uses of the metal, when reduced, by rendering that which was oxydizable in nearly all circumstances, now oxydizable in common circumstances. It makes copper much more plastic than man has made wax. It can gild with the shadow of gold, so that the million can cope with the millionaire in personal ornaments. This much has she done, and more; so much indeed, that when we contrast it with the mystical ploddings of the alchymist in his processes of transmutation of iron into copper, of copper into silver, and of that again into gold. With his "processes of melting gold in a nut-shell, dissolving gold in the palm of your hand," &c., &c., we rejoice that before our day has lived an Humphrey Davy, who could show us a metal which is lighter than, and could burn on water; and who, in his discovery, has developed a principle which has added a host of metals to the list previously known, a list now so extensive as to preclude the possibility of presenting you, in the short time I am expected to occupy your attention, with even an abbreviated notice of the simple properties and history of the various metals which form it.

Metallurgy, in its most comprehensive sense, should of right share the attention of not only the dentist, but of every enlightened man. It is a study interesting in itself, and one which would afford me pleasure to enter into in detail before you; but this is not the time for it. Estimating, as I do, your time as valuable, and applying the standard of utility to every thing we do while together, and to every thing addressed to the public eye of the profession, I cannot allow myself that freedom in diction, or that liberty with the writings of others, which, under other circumstances, it might be my privilege to do.

My purpose at the present is to direct your attention to a special division only of the great field of Metallurgy. This division I would name Dental Metallurgy, and under this head we will pass in review those metals which we daily make use of. I shall not enter on this subject after the method adopted in elementary works, such as giving the color, specific gravity, &c., of the various metals, judging that if all are not already familiar with what may be found in every system of chemistry of modern date, that he who lacks this knowledge will there seek it. My aim shall be to give you that which is not so easily arrived at, to describe more distinctly processes, which, though often treated of, lack in the descriptions given, some of the elements of success, and to add something from my experience to that of those who have written before. As gold, with the exception of a period accompanying the discovery of iron in the metallic state, has ever held the supremacy in the estimate of men, founded upon its rarity, indestructibility, and beauty, so now we yield to it a precedence in this treatise. There is one thing worthy of remark here. This is, that in dentistry has been found the first and most extensive *really* beneficial use to which gold has been put. Of nearly every other use of it, we may say as the wise man said, "all is vanity," but of our use of it we should confidently assert, *it* is the perfection of wisdom. History showeth not who introduced the arbitrary system of estimating the purity of gold by carats; but its continued adoption proves the wisdom of the arrangement. Pure gold is considered as twenty-four carats fine everywhere, except by such as know not what they talk of. The curious may find the following statements made in the Dental Journal, by a writer who attaches to his name M. D., D. D. S.: "*pure* gold, he says, is prepared by the gold beater, from twenty-two to twenty-four carats." Further he says in reference to platina, "it throws out coloring matter when acted upon by the gums;" silver also "throws out a coloring matter." Tin likewise "gives out the blue coloring matter." This I need hardly say is neither alchymical nor all-chemical; what it is I leave you to settle. For my part I wish our journals may never reflect such light again.

The quality of gold for the purposes of plate, is a subject on which exists some variety of opinion. It has doubtless been used of so low a carat, that when united by a cheap solder, and placed in mouths which have had a tendency to disease, great injury has resulted In particular emergencies it has doubtless been reduced as low as twelve carats, if not lower, by those who would be homœopathists in the use of aurum, but allopathists in the use of cuprum argentum. The rule or principle which guides them is, that the less gold they can put the patient off with, the more they have; a principle, I fear, which guides the consumer of the eighteen carat gold; whereas the principle which should guide all is, not how poor *dare* I make it, but how pure is it possible for me to make it, keeping in view the requisite elasticity which is necessary in plate work. It was guided by this principle, that when a student, I ardently, yet patiently sought for a mode by which this branch of our profession might be improved. For a time I felt very sanguine of success, and had met with much to encourage me. In fact, for a time I thought (as the inexperienced are apt to think too soon,) that I had accomplished all. What I sought was to discover a mode of doing the work, which would greatly diminish the amount of galvanic action, the result of which would be less oxydizement in the mouth, and consequently a permanently clear and bright surface.

Knowing that to produce a decided change, I must change the mode of soldering, I sought for a plan of soldering gold work with pure gold, and silver work with pure silver. This I effected by alloying pure gold for gold plate, and pure silver for silver plate, with various proportions of platina, or in some instances palladium. By these means I obtained an alloy, the melting point of which was above that of fine gold or fine silver. I have set numerous teeth on this material, but have hitherto been prevented from making a general use of it, from the difficulty of procuring teeth, the color of which will stand the heat requisite for the fusion of fine gold or silver. Where teeth of a white color are required, this mode can be adopted; but where a yellow tooth is necessary, we cannot make use of it, at least until such time as the tooth manufacturer gives us an article, the color of which is less evanescent. This is the only difficulty in the way of its general adoption, and it is one which I expect to see the skill of our manufacturers surmount as they have many others. Still following the rule of using the best article which it is possible to make use of, when the alloy of platina and pure gold suits not, I make use of gold twenty-one carats fine, the alloy consisting of twenty-one grains of gold, two copper, and one silver to the penny-weight. I prefer making use of the sovereign of the copper alloy; they are of the more recent coinage, and of course are reddest in color. In each one of full weight, there are within a small fraction, of one hundred and thirteen grains of gold, and ten grains of copper. But if the color is not red, then the alloy is most likely to be five grains of silver, and five of copper, which was the old mode of alloying British gold coin. When I use the former, or copper alloyed coin, I add five and one third grains of silver, and two-thirds of a grain of copper to each sovereign. To the same amount of the copper and silver alloyed coin, I add five and two-thirds grains of copper, and one-third of a grain of silver. Or to four sovereigns (one ounce and twelve grains when full weight,) I add twenty-one and one-third grains of silver, and two and two-third grains of copper. To the same amount of the other alloy, I add twenty-two and two-third grains of copper, and one and one-third grains silver, which will reduce either to twenty-one carats. I prefer the English coin, because I have found it purer, and consequently tougher. If German, French, American, or Spanish coin is used, they should not be further alloyed if elastic enough, as the standard of these is twenty-one. If the gold I wish to use is good, but below twenty-one carats, I add finer coin or fine gold, and thus bring it up to that carat. If it contains too much copper, or too much silver for this to be done conveniently, I refine the gold by the process called separation. My reason for using copper in excess of the silver in alloying is, it gives first, elasticity; second, a depth of color which I admire; third, by using this alloy, we obviate the necessity of removing the pickle-coat, which, instead of being of a sulphur color, presents a rich, pure gold colored surface.

Further, by using the gold thus fine, I am enabled to use solder three carats finer than he who uses eighteen carat gold, and thereby the tendency to galvanic action is diminished in the same ratio, ensuring a bright surface longer, less interference with the action of the gustatory nerve, and greater durability in the work. All parts of a piece of work to be inserted in a mouth should be of the same alloy. The clasps which pass around the teeth I leave one-third thicker than the balance of the plate. In this way I obtain the increased elasticity which is there necessary. When the gold for clasps is made of an alloy differing from the balance of the plate, it increases the galvanic action exactly where it should be most avoided, and this is a principle which shows itself even where the carat may be the same as the plate, *if* the *alloy* be varied. I make the under plate of the same alloy as the upper, depending upon the increased thickness for the necessary increase of strength. So of all the backings for the teeth, and mountings for the springs, they are all *one quality*. Unity is the rule I am guided by, and perfection is my goal, and although we are still far short of it, science will show that although the advantage may be but small in favor of twenty-one carat gold over eighteen, and of eighteen carat solder over fourteen, still it is appreciable, and therefore desirable.

In alloying, to do so correctly, it is necessary that the silver and the copper should be perfectly pure, The copper I make use of, is the best English bell wire, the toughness of which is proverbial; which property it owes to its purity. The form of it is also very convenient for cutting and weighing. When placing the copper in the crucible, care should be had that it will sink with the gold, which it is apt not to do when cut in lengths greater than the diameter of the bottom of the crucible on the inside. I generally roll it in a coil of a smaller diameter than the crucible. If this precaution be not taken, the copper is apt to remain suspended above the gold, and be oxydized and volatilized by a high heat, for contradictory as the statement may be of the books, it is a fact of which I can give the best proof, viz; That copper does melt at a higher heat than even pure gold. It is also a fact, seemingly contradictory of this, that when united to gold it reduces the melting point of that metal; but this is not the only instance in which the melting point of an alloy is less than the mean of its constituents. The silver I alloy with, is what is known among refiners as grain silver, which is generally pure. The pure silver for convenience in weighing is granulated by pouring it in a small stream into a vessel of water, hence its name. When such silver cannot be conveniently had, I would select such coin as after ignition shows little or none of the black oxide of copper on its surface. Such I

find easiest among the old Spanish coin and some of the French. These when rolled down thin may be used conveniently.

As a general thing I have little trouble in melting gold for plate. Wherever proper care is observed with the filings there may be no call for the refining process, unless, indeed, you have the misfortune to meet with a counterfeit coin, the manufacture of which is now brought to such perfection, that nothing short of separation will detect some of the issue, or remove the injurious alloy from your plate with ease or certainty if they have been added to it. The materials which most generally get into the filings and injure the plate, are lead and zinc from the metallic model and matrix, or tin when it is used for these purposes, and iron from the necessary regular and continual wear of the file. The latter may be entirely removed by the use of a small horse shoe magnet. Care should be taken to remove all oxide from the surfice of the metallic model and matrix before putting on the plate, as it is more likely to pass in, in the form of oxide than in the metallic state. They should also be kept at some distance from the lap skin. When either lead, tin or zinc is present to a large amount, the alloy should be kept in the furnace at the melting point without any flux on it, which would have the effect of protecting the base metal from being oxydized and volatilized. Where lead is present in gold, when it passes into the hands of the refiner he pursues this course, only varied by using a muffle furnace in which to heat, and a cup made of bone ash instead of a crucible of silex and clay. One or the other mode is rendered necessary when lead is present, owing to its diminished affinity for oxygen when at a low heat, (such for instance as the heat of the sand bath used in separation.) It is consequently slowly dissolved in the separating process, indeed a coating is soon formed on the gold, which partially arrests the action of the acid on the silver and copper. When tin or zinc are present in small quantities, it is not necessary to expose the alloy to the volatilizing action of the fire before separation, as their affinity for oxygen at a low heat is greater than that of lead. Another substance which will always be found mixed with our filings, is gypsum, this however presents no difficulty, when a good heat can be had, and sufficient flux is added.

The mode I pursue in melting my plate, is first to separate all the scrape of the former melting I can conveniently pick out of the filings, knowing with certainty that they are good. I next run the magnet through the filings and remove all the iron. By this picking process I gain two points; first if oxide of lead or zinc or either of them unoxydized be present, the less gold with which they are combined, the more exposed are they to the action of oxygen, and second, if any other substances be in them which would render it necessary to have recourse to the process of separating, it will require a much less amount of acid to accomplish it than if the whole mass were made inferior by adding the scraps. As a flux, I add to every five or ten dwts., of filings as much pulverized nitre as will lay on a quarter dollar, these I thoroughly mix and place in a small crucible.

The scraps and additional coin I place with a small piece of borax in an other crucible, and place both in the fire at the same time. After the filings are perfectly melted, I either pour the gold into an ingot, or allow it to cool in the crucible, which I then break and test the gold by a few hard blows, which if it stands without cracking, is of course fit to be added to that in the other crucible. If however it is not good enough and I think that by fluxing I can make it so, I place it in a clear crucible and try the effect of heat without flux, which I follow by adding flux after one hour's steady heat. If this does not suffice, I lay it to one side to be purified by acids, considering the labor of the humid process much easier than that of the dry, when a piece of really coarse gold falls into my hands. It has also the advantage of being the only certain mode of arriving at the desired effect.

In melting gold and silver, much depends upon having a good heat. To procure this I prefer using the draft furnace. This is one in which the bellows are dispensed with, and the necessary air for the combustion is supplied by the draft of a good chimney. These furnaces are generally built of brick, their form is commonly square, the space for the fuel may be six inches square, and nine inches in depth. For the last three years I have made use of a furnace somewhat similar to the tooth furnace, which I much prefer for many reasons. The length of this article advises me not to enter here into a minute description of either form; but should a fitting opportunity present I may enter more into detail.

The proper fuel for melting gold is coke, which when good is solid, heavy, and of a steel grey color. It is particularly adapted where a long continued heat is required. The proper fuel for silver is charcoal, owing to that metal's great affinity for sulphur, prohibiting the use of coke. The best charcoal made in the west is formed of the elm and sugar tree timber. When charcoal is used for a long heat, there should be placed on the bars of the furnace a piece of fire brick two inches square, on which the crucible should rest. If it is a small one, it is safest to set it in the lower half of a large one, both of which should be set on the brick. This allows of the removal of the fuel without shifting the crucible. The cover of the crucible should never be iron, as scales of its oxide are apt to drop into the gold, the bottom of a crucible or another reversed makes a better cover. There are two kinds of sand crucibles brought to market. One, which is of a bluish shade and partially vitrified, showing there is present a goodly portion of oxide of iron in the clay used in their manufacture; owing to this they do not stand the variations of temperature as well as those made from a purer clay. The latter kind are of a yellow or brown shade, and have my decided preference.

There is no call for the black lead crucible in our operations. They are of advantage only when a constant melting is kept up, they being capable of standing seven days steady heat of a powerful furnace, but support poorly the variations from hot to cold, or the reverse. They are also easily decomposed by an alkaline flux.

I have elsewhere spoken of separation. This is one of the modes of purifying gold, and the one which I would say, (knowing thoroughly all other modes,) is best suited to the dentist's use in rendering pure a piece of inferior gold. It is the mode by which in general gold is prepared by the professional refiner for use in the various branches of art to which pure gold is applied. The process is based upon the property possessed by gold of resisting the action of nitric or sulphuric acids, while the baser metals with which it is usually adulterated are dissolved by either. Separation by means of nitric acid is comparatively ancient, while that by sulphuric is quite modern. Refining by means of sulphuric acid was introduced by M. Diz, while inspector of the French mint some years ago. By its means, some mammoth establishments in Paris retain the business of refining nearly all the bar silver which passes from both Americas, the deposites in some of the mines being aurarets. Its chief excellence is said to consist in its power of separating a quantity more minute than can be effected with the nitric acid. This I consider doubtful. By means of the cheapness of sulphuric acid, combined with the perfect arrangements of very costly apparatus which returns to them nearly all the acid, together with their forming all the copper dissolved from the alloy with the commercial sulphate, the Parisian refiners are enabled to refine large quantities of silver, rich in copper, and receive only the copper as a remuneration; to such perfection have they brought this branch of art. Still, although possessed of these and some other advantages, it is not in any way calculated for our purposes. The apparatus necessary for the nitric acid process is simple and cheap, and so far as the purity of the gold is concerned, is equally successful. The process consists, first in melting the gold with three times its weight of silver. Less than this amount, say twice its weight of silver, may answer when the gold contains five or six dwts. of copper in the ounce. While this alloy is in a state of fusion, it should be stirred with a rod of fire clay, previously brought to a red heat, else gravitation will be found to have made one portion of the alloy richer than another, and consequently make the separation difficult. Immediately after this, it is to be removed from the fire, and granulated. This is commonly effected by pouring in it a small continuous stream into a large tub of water, moving the crucible round in a circle, to prevent the grains adhering. Some years since, I introduced a much more convenient and perfect mode of performing this operation. I make use a common sized wooden pail which I fill with water, above it is placed another without a bottom, its end fitting tightly into the top of the lower one. About three inches above the water, I bore four holes through the sides of the upper one. In these are fitted tightly four round pieces of wood, (½ inch in diameter,) these project into the interior of the vessel, and form a support for two or three tiers of round wood, (old rose-bush branches are best,) crossed on each other so as to form meshes, none of which are opposite each other. On to this the metal is poured from a height of about 18 inches. The result is an extreme minute division of the metal, which allows of a quicker and more perfect separation of the alloy. The granulated alloy is next placed into a bottle, technically known as a flask, no part of which should be much over 1-16 of an inch thick, that it may stand the changes of temperature. The bottom should be nearly flat, from which the upper portion should rise in the form of a funnel, the mouth being 1½ inch in diameter. The metal may be easily removed from the bucket and slid into the flask with a spoon. To accelerate the action of the acid, use is made of a sand bath; a convenient one may be made of a small shallow iron saucepan, in which place an inch of sand, in this the bottom of the flask is to be set; the heat may be applied by means of a charcoal furnace.

The inexperienced in refining are frequently baffled by procuring an inferior article of acid. For refining, the acid should be pure. In particular, it should be free from muriatic acid, with which the aquafortis commonly sold as nitric acid is mixed. It also frequently contains sulphuric acid, from both of which it may be separated. The first must be removed before refining can proceed; the second presents little difficulty. The test to show the presence of and a reagent for the removal of muriatic acid, is a solution of silver in nitric acid, or a solution of lunar caustic in water. When this is added, if muriatic acid be present, there is instantly formed an insoluble chloride which is precipitated; the reagent should of course be added slightly in excess. The test for sulphuric acid, is a solution of the muriatic of baryta; when this is added, if sulphuric acid be present, the sulphate of baryta is immediately formed. The method of purifying acid on the large scale, is to re-distill the crude article. The nitric acid should not be placed on the alloy of the full strength at first, but should be used in the proportions of two parts acid to one of water, for the first two portions; the third may be used the full strength. It is customary in text books to name the time the successive portions of acid should remain on the gold; instead of this, I would advise its being kept on as long as there is any action present, which is known by the disengagement of nitrous oxide which escapes in the form of red fumes from the matrass. When this ceases renew the acid, unless when it ceases from the silver being all dissolved and the gold consequently purified, which fact is known by its presenting a uniform dark brown color, which is raised to the true gold color by annealing.

Care should be taken in pouring off the acid to allow only the clear saturated solution to escape, as at times some portions of gold will become so comminuted as to float in it when agitated.

For the precipitation of the silver from the solution, there are two modes practiced. One is, to precipitate by means of sheet copper; in the other, by means of muriate of soda, (common salt.) For either process, the solution is diluted by adding twenty parts of water to one of solution. In the first process, the silver is deposited in the metallic state; in the other, it is thrown down in the form of a chloride. The proper vessel for the first mode, is a stone-ware jar; into this, place the dilute solution, and in it place a piece of stout sheet copper as long as the jar and two or three inches wide. The silver will soon be seen to attach itself to the copper, and as it accumulates it will slip to the bottom, exposing the copper for a new deposite, until all which it will remove has been precipitated. There is still a small trace of silver in it, which is thrown down in another vessel in the form of chloride by means of the salt, muriate of soda being the most delicate test for silver we possess. The chloride in this second vessel I allow to accumulate until there are a few ounces, when I bring it to the metallic state by a process to be described. The silver precipitated by the copper may be rendered perfectly pure by throwing it on a filter and washing with warm water until all traces of acid leave it; it may then be melted with borax and granulated, when you will have a supply of the best silver for the purposes of alloying which can be had. If you should still fear the presence of acid in the silver, melt with salamoniac, the alkaline properties of which will neutralize the acid. If it is not convenient to procure copper, you may adopt the chloride mode. For this purpose a wooden vessel, (in the absence of a stone one,) will answer. In it first place the requisite amount of water required in diluting; in this dissolve the salt in excess; you may then pour in the silver solution, when the insoluble chloride is immediately formed. When the precipitate has fully settled, the liquor may be run off with a syphon, before doing which it should be tested with salt. The chloride should next be thrown on a filter, and the nitric acid washed out with warm water; it may next be thrown back into the vessel or a small stoneware dish. Into the wet chloride must now be placed zinc, either in the form of granules or strips, (the latter I prefer,) a little sulphuric acid is then to be poured into the vessel and mixed by stirring. The decomposition of the zinc with the liberation of hydrogen gas which results, effects the decomposition of the chloride of silver which it leaves in the metallic state. The chloride is known to be all decomposed, when it is entirely changed to a dirty yellow color, the former being pure white, if not subjected to the action of light. Care should be taken that all the zinc is dissolved, before melting the silver; for this purpose, the sulphuric acid should be used in excess. The silver is next to be thrown upon the filter, and the sulphate of zinc washed from it; it is then ready to be melted with borax or salamoniac. Another process, which may be convenient for a small amount, is, to allow the washed chloride to lay on the filter until it dries; with it mix one-third its weight of pulverized carbonate of soda; it is then to be placed in a crucible and reduced in the furnace.

5

If at any time it is desirable to test the quality of a piece of gold plate, the easiest mode of doing so, is to take twelve grains of it, to which add thirty-six grains of silver, and melt them on charcoal with the blow-pipe, the resulting button may then either be hammered or rolled out thin and placed in a thin two ounce vial, or small flask if this is convenient. The acid may then be applied as before described. If you have bought as eighteen carat, gold which is really so, you will have as the result of your experiment nine grains of pure gold, provided you have conducted the operation with proper care. Whatever the amount is which a half dwt. yields, double that and you have the carat of the gold. If gold foil has been poorly purified, the presence of the smallest trace of silver may be detected by dissolving a leaf in nitro-muratic acid, if there, it will be found at the bottom of the vessel in the form of chloride.

Of the remainder of dental metallurgy, I am pleased to say little need be said, the more so as this essay already far exceeds the limits within which I expected to embrace the few thoughts here presented.

As to whether there should be one or two metals used in forming casts, and what these should be, there exists (it may be with good reason) some variety of opinion. Some there are who consider block tin suitable both for model and matrix; others prefer the type metal matrix and zinc model, while the great majority make use of a lead matrix and a zinc model. This last has my own preference, I use the zinc model for its hardness, and the lead matrix because it adapts itself to the model. Whichever mode is adopted, the metal should be stirred and the oxide removed. This is of particular advantage to the zinc. When first melted from the block it should be stirred with a stick, the combustion of which is of benefit in purifying it from the oxide which forms in its interstices which result from crystalization. If type metal is desired, it may be made after the common mode by adding to your lead one third its weight of antimony. Leaving these we will pass to the last of the metals we shall now consider.

Throughout the entire circle of our operations we are constantly reminded of the importance of having instruments constructed of good steel, and properly tempered. And the fact, that we are frequently compelled to repoint excavators to adapt them to peculiar cases, necessarily forces us to acquire a knowledge of the manufacture of steel, and the best modes of tempering it. Steel you are doubtless aware is a carburet of iron, the compound being formed by placing bar iron imbedded in ground charcoal in an oven shaped furnace, and raising the heat gradually to 100° Wedgwood at which it is kept for six or eight days. There are three kinds of steel known in commerce, under as many different names. Blistered steel is the carburetted bar iron just as it comes from the furnace, and presents on its surface numerous blisters, which are attributed to the bursting of vesicles of carbonaceous matter. The next quality is known under the name of *shear* steel, from the fact of its being used in the manufacture of shears for dressing cloth. It is formed by binding several bars of the blistered steel together, heating to the welding point, and subjecting them to a process of tilting, which condenses the particles, from which results greater toughness, and a susceptability for a higher polish. The third kind has been introduced at a comparatively recent date. It is known under the name of cast steel, and is used in the manufacture of the best cutlery, surgical and dental instruments, fine files, and the best mechanical tools. In its manufacture, the blistered steel is broken into fragments, which are placed into a crucible and melted and cast into ingots, hence its name. It is susceptable of a higher polish than the shear steel, but must be worked at a lower heat. A good article of cast steel, when purchased, presents a fine grained surface, entirely similar throughout. The addition of 1-500 of silver to steel, is said to render it susceptable of a higher polish and keener edge.

The addition of Iridium and osmium to iron, forms a compound, which may be tempered, and is less liable to oxydize than iron or steel. In working steel, an important point, is to make it as soft as it possibly can be made before filing; this is best effected by making a fire of soft wood over the objects to be softened, and allowing them to remain in the ashes until perfectly cold. The mode I adopt for repointing an excavator, is to heat it in the flame of an alcohol lamp, and by means of a pair of very long beaked pliers, also heated, I bend it to the desired shape while in the flame. In hardening and tempering steel, water is most generally used; some cutlers prefer oil, while some die sinkers, make use of naphtha, which having no oxygen in its composition, the chances of injury are somewhat diminished. The mode I pursue, is to heat an inch of the point of the excavator to a bright red (generally by means of the blowpipe,) and quench immediately in cold water. This, if the steel be good, makes the point nearly as brittle as glass; it requires next to be tempered. In tempering steel, the workman is guided by certain successive colors, which a bright piece of steel assumes when subjected to a gradually increasing heat, each of which colors indicates a different degree of hardness, and he with certainty selects the temper suited to the work, by selecting the color indicative of it. By some operators, nine different colors are distinguished. They have four shades of yellow, from a faint to a brown yellow, two of purple, and three of blue. If we resolve these into three shades, I think we will be in possession of the variety required by the dentist. These would be a bright yellow, a purple, and a full blue. The first for large pointed excavators and scalers, the second for small ones, and the third for pluggers and forceps. After hardening, the next step is to polish the point so as to show the changes of color, (the easiest mode for the dentist is to rub it on the oil stone.) The instrument I then place in the flame of the lamp, allowing the polished portion to project beyond it. Close by the flame stands a tumbler of water, into which the instrument may be instantly plunged, as soon as the desired color presents. The color of course shows itself nearest the flame first, and gradually reaches the point, which is the spot the eye should rest on. The first color assumed is the yellow, (the fainter the yellow the harder the instrument;) next the purple, followed by the light blue, the full, and the dark blue.

Such, gentlemen, although a somewhat tedious, is nevertheless a very condensed view, not of the entire subject allotted to me, but simply a division of it. My desire is, that there may be something contained in it which will prove of value to you. If this is gained, my object is attained.

